# Low frequency of asymptomatic dengue virus-infected donors in blood donor centers during the largest dengue outbreak in Taiwan

**DOI:** 10.1371/journal.pone.0205248

**Published:** 2018-10-08

**Authors:** Jih-Jin Tsai, Ping-Chang Lin, Ching-Yi Tsai, Ying-Hui Wang, Li-Teh Liu

**Affiliations:** 1 Center for Dengue Fever Control and Research, Kaohsiung Medical University, Kaohsiung City, Taiwan; 2 Tropical Medicine Center, Kaohsiung Medical University Hospital, Kaohsiung City, Taiwan; 3 Division of Infectious Diseases, Department of Internal Medicine, Kaohsiung Medical University Hospital, Kaohsiung City, Taiwan; 4 School of Medicine, College of Medicine, Kaohsiung Medical University, Kaohsiung City, Taiwan; 5 Department of Medical Laboratory Science and Biotechnology, College of Medical Technology, Chung-Hwa University of Medical Technology, Tainan City, Taiwan; University of Hong Kong, HONG KONG

## Abstract

To determine the prevalence of asymptomatic dengue virus-infected blood donors during the largest dengue outbreak in Taiwan history occurred in 2015, we examined the evidence of dengue virus (DENV) infection by the detection of DENV RNA genome using real-time reverse transcription-polymerase chain reaction (real-time RT-PCR), DENV NS1 antigen using rapid diagnosis test (RDT) and anti-dengue antibody using IgM/IgG capture enzyme-linked immunosorbent assay (capture ELISA) and RDT in eight thousand serum samples from blood donations to the blood centers of the Taiwan Blood Services Foundation (TBSF) in Kaohsiung City and Tainan City during the largest dengue outbreak in Taiwan history occurred in 2015. Only one serum sample was positive for DENV RNA detection by using dengue-specific real-time RT-PCR, the virus was DENV-2 determined by serotype-specific real-time RT-PCR and sequencing, and the DENVs in the serum were confirmed as being infectious by a plaque assay. The recipient of this blood did not develop any dengue fever symptom on follow-up. None of the samples was NS1 RDT-reactive. Seventeen IgM-positive samples were identified. There was a low prevalence of asymptomatic confirmed or probable DENV-infected blood donors in our study (0.013% and 0.21%, respectively), and no symptomatic transfusion-transmitted dengue (TT dengue) was developed during the largest dengue outbreak in Taiwan history in highly endemic areas and periods.

## Introduction

Dengue is an arthropod-borne viral disease, which produces a wide spectrum of clinical symptoms and outcomes, mainly occurs in tropical and subtropical areas. This disease develops following infection by the dengue virus (DENV), which is an RNA virus with an approximately 11-kilobases positive-sense RNA genome. Dengue viruses are transmitted to humans by the bites of DENV-carrying mosquitoes, especially *Aedes aegypti* and *A*. *albopictus*. DENV infections result in asymptomatic infections, undifferentiated fevers, dengue fever (DF), dengue hemorrhagic fever (DHF) and dengue shock syndrome (DSS) [1]; alternatively, they can be classified as dengue with warning signs, dengue without warning signs, and severe dengue [2].

The first dengue outbreak in Taiwan since World War II occurred in 1981 (~8,000 cases, DENV-2) [3]. Over the past three decades, there have been four subsequent large dengue outbreaks (>4000 cases), happened in 1988 (4,389 cases, DENV-1), 2002 (5,388 cases, DENV-1 and DENV-2), 2014 (15,732 cases, DENV-1) and 2015 (43,784 cases, DENV-1 and DENV-2), over the past three decades [4–8](S1 Fig). DF cases in the other 26 years ranged from 10 to 2,179 (mean 573.15, 95% CI 318.30–828.01, median 327, 95% CI 142.29–689.79). Dengue in Taiwan usually starts with imported cases from Southeast Asian countries [9], spreading during the rainy and warm months starting in July, then peaking in October or November, and finally declining when the weather becomes cooler. Accumulated data between 1998 and 2017 revealed DF cases clustered in Kaohsiung City (58.5%), Tainan City (34.0%) and Pingtung County (2.8%) in Taiwan. Among these DF cases, 3.98% were imported cases, while the rest were autochthonous cases.

It is estimated that 50–85% of DENV-infected people have asymptomatic infections, and these asymptomatic people can contribute to DENV transmission since they may be exposed to more mosquitoes through their undisrupted daily routines than sick people [10]. These asymptomatic DENV-infected people might also transmit DENV to other people through blood donation and transfusion [11–13]. Moreover, many studies have reported that DENV was detected in donor blood in blood centers in Honduras, Brazil, Australia [14], Puerto Rico [15] and Saudi Arabia [16] using nucleic acid tests (NAT), such as transcription-mediated amplification (TMA), reverse transcription–polymerase chain reaction (RT-PCR), and antigen/antibody tests. The recipients of DENV-contaminated blood components might contract transfusion-transmitted dengue (TT dengue) [11–13, 17].

Dengue is a notifiable infectious disease in Taiwan determined by the Centers for Disease Control, Taiwan (Taiwan CDC). Human serum samples of suspected dengue cases nationwide must be submitted to the Taiwan CDC or a Taiwan CDC-certificated dengue diagnosis laboratory, Tropical Medicine Center (TMC) of Kaohsiung Medical University Hospital (KMUH) in Kaohsiung City, Taiwan, to confirm DENV infection [6]. Consecutive large dengue outbreaks in Taiwan in 2014–2015 urge us to face the safety of blood transfusion, especially in DF endemic seasons. In 2015, over ninety-seven percent of DF cases were occurred in Tainan City (22,777 cases) and Kaohsiung City (19,784 cases), which are located south of the Tropic of Cancer (E120°~E121°), where *A*. *aegypti* is the dominant mosquito species (S2 Fig). There were 106,992 blood donations and 109,511 blood donations to the Kaohsiung City blood center and the Tainan City blood center, respectively, at the time of the dengue outbreak that occurred between September and November in 2015. Additional blood is routinely collected from donors and are stored frozen in a repository of the Taiwan Blood Services Foundation (TBSF) in Hsinchu County located in Northern Taiwan for pathogen surveillance and confirmation testing once transfusion-transmitted infection was suspected. To investigate the prevalence of blood donors with recently asymptomatic DENV infection, we conducted a retrospective study to detect the presence of the DENV RNA genome, NS1 antigen and anti-dengue IgM/IgG in bloods that were donated to the blood centers of the TBSF in Kaohsiung City and Tainan City during the largest dengue outbreak in Taiwan history in 2015.

## Materials and methods

### Human serum samples and ethics statement

Under the entrustment and authority from the Taiwan CDC (please refer to the Financial Disclosure for the funding by Taiwan CDC to JJT and LTL) and the help of coordination with the TBSF, we obtained eight thousand de-identified residual serum samples collected in serum separation tubes (SST, Becton Dickinson, Franklin Lakes, NJ, USA) (4000 from each center), which were randomly selected from the outbreak districts of Kaohsiung City and Tainan City that were collected during the outbreak period, from the staffs of the TBSF and tested for the evidence of DENV infection at the TMC dengue diagnosis laboratory, which is an ISO 15189:2012 certified dengue diagnostic laboratory certificated by the Taiwan CDC, the Taiwan Accreditation Foundation (TAF) and the International Laboratory Accreditation Cooperation (ILAC) Laboratory Combined Mutual Recognition Arrangement (MRA) Mark. The study was reviewed and approved by the Institutional Review Board of Kaohsiung Medical University Hospital (KMUHIRB-EXEMPT (II)-20160009) and waiver of informed consent was obtained because the study play a role in the control and the prevention of dengue fever in Taiwan.

### Viral RNA extraction and one-step SYBR green-based real-time RT-PCR for the detection of DENV genome in the pooled samples

For the detection of DENV genome in pooled sera, viral RNA was extracted from 200 μL of the pooled serum (combined ten single serum aliquots into one pool) or the control serum by using the PureLink Viral RNA Mini Kit (Life Technologies; USA) and immediately subjected to real-time RT-PCR analysis. Dengue-specific primers for the DENV RNA detection were DN-F: CAA TAT GCT GAA ACG CGA GAG AAA and DN-R: CCC CAT CTA TTC AGA ATC CCT GCT. Serotype-specific primers for molecular serotyping were DN-F: CAA TAT GCT GAA ACG CGA GAG AAA, D1-R CGC TCC ATA CAT CTT GAA TGA G, D2-R: AAG ACA TTG ATG GCT TTT GA, D3-R: AAG ACG TAA ATA GCC CCC GAC and D4-R: AGG ACT CGC AAA AAC GTG ATG AAT [18]. These primers amplified the genomic region encoding the nucleocapsid or core protein. Real-time RT-PCR was performed in a Mx3000P machine (Agilent/Stratagene, USA) by using the Brilliant II SYBR Green QRT-PCR Low ROX Master Mix system (Agilent/Stratagene, USA). Amplification plots and Tm values were analyzed to verify the specificity of the amplicon.

### RNA extraction and one-step SYBR green-based real-time RT-PCR for the detection of DENV genome in serum from single donors

For the detection of DENV RNA genome in single serum from eight thousand blood donors, viral RNA was extracted from 200 μL of the serum by using the TANBead Nucleic Acid Extraction Kit in Smart LabAssist-16 Automated Extraction Instruments (Taiwan Advanced Nanotech Inc., Taoyuan, Taiwan) and immediately subjected to real-time RT-PCR analysis. Dengue-specific primers for the DENV RNA detection were the same as described above. Real-time RT-PCR was performed in a Mx3000P machine (Agilent/Stratagene, USA) by using the One Step RT-QGreen 2X SybrGreen Low ROX Master Mix system (CellSafe, Gyeonggi-do, Republic of Korea). Amplification plots and Tm values were analyzed to verify the specificity of the amplicon.

### Preparation of dengue viruses

Four serotypes of DENV, including DENV-1 (US/Hawaii/1994 strain), DENV-2 (New Guinea C strain), DENV-3 (DN8700829A strain) and DENV-4 (DN9000475A strain), were propagated in C6/36 cell line. The stock viruses was diluted in RPMI medium containing 1% fetal calf serum (Gibco-Life Technologies, USA) and added to the C6/36 cells with a final titer of 0.01 multiplicity of infection of. Culture fluids were harvested after incubation for 4–7 days (28°C 5% CO_2)_ until cytopathic effect was observed. Culture supernatants were used as positive control for real-time RT-PCR and as viral antigen in anti-dengue IgM/IgG capture ELISA.

### Plaque assay

DENV titer of culture supernatants and serum from blood donor was determined by plaque assay. Briefly, fresh DENV culture supernatants and serum from blood donor were 10-fold serial diluted in the MEM medium (Gibco-Life Technologies) and added to monolayer of Vero cells in 6-well plates at a density of 1 × 10^6^ cells per well. DENV adhesion onto Vero cells was allowed at 37°C, 5% CO_2_ for 2 h, medium containing DENV was removed before 3 ml overlay medium containing 1.2% methylcellulose was added. Vero cells were incubated for 5–10 days further until plaques became apparently visible by microscopy examination, fixed, and stained with crystal violet. Plaques were counted manually and plaque forming units (PFU) per ml were determined [19].

### Detection of DENV NS1 and anti-dengue IgM/IgG by RDT

DENV NS1 and anti-dengue IgM/IgG in serum samples were detected using SD BIOLINE Dengue Duo (STANDARD DIAGNOSTICS, INC., Korea). All RDTs were performed and the results were determined according to the manufacturer's instructions. In brief, for DENV NS1 antigen, approximately 100 μL of serum were added into the sample pad and tests results were interpreted after 15–20 min. Similarly, for anti-dengue IgM/IgG, 10 μL of serum was added into the sample pad and 90–120 μL of assay diluent were then added to the diluent well. Test results were interpreted after 15–20 min.

### Detection of anti-dengue IgM and IgG by ELISA

Anti-dengue IgM and IgG of all serum samples were tested at 1:100 dilution by capture ELISA using the detailed experimental procedures described elsewhere [20]. The optical density was determined at 405 nm with a reference at 630 nm in a μQuant Model MQX200 Microplate Spectrophotometer (BioTek Instruments, Inc., USA). To exclude the cross-reaction of the anti-Japanese encephalitis virus (JEV) antibody in the assays, culture supernatant from JEV-infected Vero cells was used as negative control antigen routinely. A positive sample was defined as having a test absorbance≥0.5 and a ratio of the positive control to the negative control≥2.0, and a negative sample was defined as having a test absorbance<0.5.

## Results

### Sample collection strategy and donor information

**Fig 1:**
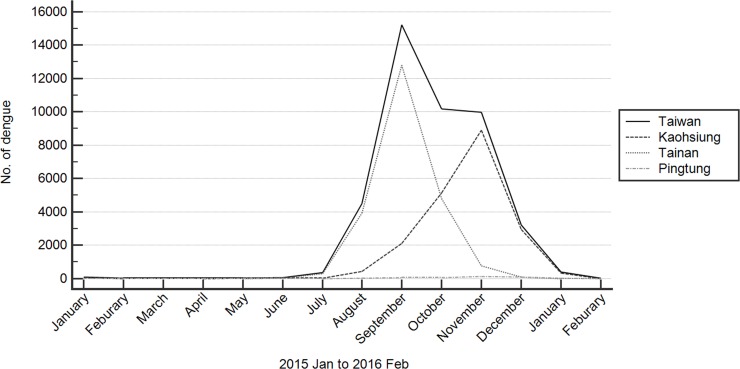
Monthly dengue fever cases in three southeastern cities in 2015. Monthly confirmed dengue case numbers are shown in three southeastern cities that were the three cities with the highest number of dengue cases in Taiwan’s history. We surveyed the presence of the DENV in the blood collected in the blood centers of Kaohsiung City and Tainan City.

Eight thousand de-identified serum samples that had been collected during the most active dengue outbreak period from 30th August to 26th September in Tainan City and from 4th November to 25th November in Kaohsiung City (Fig 1) were randomly selected for analysis. The information available from the TBSF about the blood donors in this study was shown in Table 1; all donors met the criteria for blood donation suggested by the TBSF at the time they donated blood.

**Table 1:** Background information of the blood donors.

Age	Tainan City Blood Center					Kaohsiung City Blood Center				
	Male		Female		Subtotal	Male		Female		Subtotal
	n	%	n	%		n	%	n	%	
<20	65	2.48	112	8.15	177	33	1.28	75	5.29	108
20–29	512	19.5	467	33.99	979	488	18.91	413	29.11	901
30–39	783	29.82	377	27.44	1,160	790	30.61	385	27.13	1,175
40–49	647	24.64	211	15.36	858	676	26.19	243	17.12	919
50–59	498	18.96	169	12.3	667	465	18.02	237	16.7	702
>60	121	4.61	38	2.77	159	129	5	66	4.65	195
Total	2,626	100	1,374	100	4,000	2,581	100	1,419	100	4,000

### Detection of the DENV RNA genome in the pooled serum samples

We performed dengue-specific real-time RT-PCR using Brilliant II SYBR Green QRT-PCR Low ROX Master Mix system to detect the presence of DENV RNA genome in eight hundred pooled serum samples (ten single donations per pool). Only one serum pool was positive according to dengue-specific real-time RT-PCR in the screening. The result was confirmed as being positive, and then the ten single serum samples in the pool were subjected to another round of dengue-specific real-time RT-PCR. One serum sample was positive according to real-time RT-PCR (case no. 1142 in Table 2).

**Table 2:** Summary of the analysis of eight thousand blood donations collected during the 2015 dengue outbreak.

			RDT & ELISA^b^	
Case No.^a^	RT-PCR	NS1	IgM	IgG
695	-	-	+	+
1142	+	-	-	-
1368	-	-	+	+
1830	-	-	+	-
1946	-	-	+	-
1981	-	-	+	+
1986	-	-	+	+
1993	-	-	+	+
2204	-	-	+	+
2232	-	-	+	-
2434	-	-	+	-
2510	-	-	+	+
2642	-	-	+	+
3659	-	-	+	+
4975	-	-	+	+
7371	-	-	+	+
7520	-	-	+	+
7528	-	-	+	+
Positive	1	0	17	13
Negative	17	18	1	5

^a^ Only the cases with a positive result are shown in the table.

^b^ Results of the combination of the RDT and ELISA.

### Molecular serotyping, sequencing and infectious titer of dengue virus in donor serum

To understand the serotype of the DENV detected in the donor serum, serotype-specific real-time RT-PCR was performed. The result of the serotype-specific real-time RT-PCR indicated the DENV in this sample was DENV-2 (case no. 1142 in Table 2). The results of the serotype-specific real-time RT-PCR was confirmed by sequencing of the DENV in this serum. To understand the infectious potential of this DENV positive serum, we performed the plaque assay to measure the titer of infectious DENV, which might bring out TT dengue, in this serum. The result of plaque assay indicated that the titer was equivalent to 50 PFU/ml.

### Re-evaluation of the presence of the DENV RNA genome using single serum from blood donors

The surveillance of DENV genome using pooled-sample strategy revealed only one donor serum was DENV RNA positive. To investigate if any DENV-positive sample was missed by using pooled-sample strategy, we later re-evaluated the presence of the DENV RNA genome using single serum from eight thousand blood donors. The results of this re-evaluation study indicated there was only one donor serum positive for DENV real-time RT-PCR (case no. 1142 in Table 2).

### Dengue NS1 antigen and anti-DENV IgM/IgG detection

The presence of NS1 in the serum is one of the confirmed dengue diagnostic criterion [2]. Thus, we detect the presence of DENV NS1 antigen using SD NS1 RDT. The results indicated that none of the samples were NS1-reactive (Table 2). Next, we analyzed the anti-dengue antibody response of these donors to the recent DENV infection by using IgM/IgG capture ELISA and RDT. We combined the results of ELISA and RDT to maximize the possibility to detect anti-dengue antibody presented in donor’s sera. Seventeen cases were IgM-reactive according to ELISA or RDT (0.21%) (Table 2). In these cases, thirteen cases were IgG-reactive (0.16%).

## Discussion

The primers used in this study were based on highly conserved region of the gene encoding the nucleocapsid or core protein of dengue virus genome, and were able to detect all four DENV serotypes. The detection limit of the in-house DENV real-time RT-PCR used in this study was tested using 10-fold serial dilutions of dengue virus culture supernatants (using RPMI medium containing 10% FBS) that had been previously quantitated by plaque assay. The detection limits of the standard serotype-specific primer pairs were 2.5 PFU/ml for DENV-1, 2.1 PFU/ml for DENV-2, 10 PFU/ml for DENV-3, and 19 PFU/ml for DENV-4. In vitro quantification of dengue genomes of supernatants were then performed using Dengue Virus subtypes 1, 2, 3 and 4 genesig Standard Kit (sensitive to < 100 copies of target) (please refer to Supporting methods). The quantification results of dengue virus supernatants suggested that the detection limit of the in-house DENV real-time RT-PCR were approximately equivalent to 1500 copies/ml for DENV-1, 1050 copies/ml for DENV-2, 2000 copies/ml for DENV-3, and 3800 copies/ml for DENV-4.

Our real-time RT-PCR results demonstrated only one DENV viremia case among the eight thousand blood donations collected during the 2015 DF outbreak in southern Taiwan (0.013%). The virus could be cultured from this real-time RT-PCR-positive serum sample, suggesting that a risk of TT dengue existed. The virus was DENV-2 which was confirmed by serotype-specific real-time RT-PCR and sequencing of the RT-PCR products. The virus was confirm to be the cosmopolitan genotype using the phylogenetic analysis of the envelope gene sequence using the methods described in our previous article [21]. The phylogenetic analysis result was similar to the result by Wang et. al.[8]. After reporting this case to the TBSF and the Taiwan CDC in May 2016, the recipient of this blood was contacted for follow-up about seven months later after transfusion. The recipient did not develop any dengue fever symptom after transfusion and thus no symptomatic transfusion-transmitted dengue case was developed in this study. We did not get access to the serum of both the DENV-positive donor and the recipient of the DENV-positive unit on follow-up at that time, one of the major reason was it is out of the scope of the original study approved by the IRB. The donor of this DENV-positive blood was possibly in an early stage of asymptomatic primary DENV infection (Table 2).

The infectiosity of DENV had been study extensively in cell models. The infection of DENV exhibits a dose-response manner both in cell model with [22] and without [23] the presence of anti-dengue antibody. In humans, the situation are supposed to be more complicated due to the variation of the immune status and susceptibility in different individuals. According to the statement of Stramer et. al., the infectious dose of DENV by transfusion is largely unknown [13]. No association between donation viral load and transmission to recipients was evident had been reported. It is suggested that the infectious dose required for transfusion transmission may be higher than expected, since 16 RNA-positive units transfused into 16 susceptible recipients only resulted in 5 cases which were considered probable TT cases (the TT rate was 37.5% in their study) [17]. We assumed the reasons why the recipient of the infectious DENV-positive blood in this study did not develop TT dengue can be 1. The virus load is not enough to initiate an effective infection, 2. The low susceptibility to the DENV infection of the recipient, 3. Developed immunity from previous DENV infection against current DENV infection, and 4. It was asymptomatic infection since the recipient did not developed any DF symptoms after transfusion.

The DENV NS1 protein is a 44–49 kilodalton glycosylated protein which actively participates in viral RNA replication. The circulating NS1 level was 10 ng to 50 μg/ml in the blood during the acute phase of DENV infection determined by ELISA and it is suggested that DENV NS1 could be detected even when viral RNA was negative in real-time RT-PCR or in the presence of IgM antibodies [24]. Furthermore, the detection of the presence of NS1 in the blood is one of the dengue diagnostic methods [2]. However, the sensitivity of NS1 RDT was ranging from 44.4% to 87% [25–28] in the detection of DENV when compared to real-time RT-PCR. Besides, the circulating level of NS1 in asymptomatic DENV infections has not been determined. None of the samples tested in this study was NS1 positive. Taken these together, the detection of NS1 might not be useful for DENV screening in bloods from asymptomatic donors at this moment.

In this study, we found seventeen IgM-positive samples (thirteen were IgG positive among these samples) which were real-time RT-PCR negative. The presence of anti-dengue IgM antibody in a single serum sample was recognized as a probable dengue case according to the definition by World Health Organization [2]. So there were seventeen blood donors which were asymptomatic probable DENV-infected volunteers (0.21%). Although the seroprevalence of blood donors had been documented [14–16, 29–35], the impact of the IgM positive- or IgM/IgG dual positivity bloods on the transfusion transmission of DENV is largely unknown today despite it has been proposed that not only DENV but also anti-dengue antibodies may pose a risk to blood transfusion safety [36].

The prevalence of asymptomatic dengue viremia among people aged 20 to 64 years was estimated to be 15.0 per 10,000 (0.15%), by using an established mathematical model using some parameters and based on assumptions, in Tainan during the 2015 epidemic [37]. Conversely, our results showed a relatively low frequency of the presence of DENV RNA genome in the asymptomatic blood donors (0.013%). If their estimation was correct, then there might be some reason the detection rate of DENV RNA genome was relatively low in our study. One limitation of our study is that the detection method of DENV RNA we used was real-time RT-PCR, which is not as sensitive as the TMA or enhanced TMA used in other reports [14, 15, 38]. In addition, the presence of PCR inhibitor in the bloods of donors might confer to the inhibition of real-time RT-PCR in our study. Furthermore, seventeen probable DENV-infected donors might in the antibody response phase of the infection, which the virus might decline to the level below detection limit of real-time RT-PCR used in this study.

To our knowledge, this is the first study to survey the prevalence of asymptomatic confirmed and/or probable DENV-infected blood donors in the dengue endemic time periods in the blood centers in Taiwan. The prevalence of the DENV RNA, NS1 antigen and anti-dengue antibody in blood donors in highly endemic cities in this study was relatively low compared to that in other countries or areas such as Honduras[14], Brazil [14, 38, 39], Puerto Rico [15], Guangzhou [35], Saudi Arabia [33] and India [40](S1 Table).

## Conclusions

There was a low prevalence of asymptomatic confirmed or probable DENV-infected blood donors in our study (0.013% and 0.21%, respectively), and no symptomatic TT dengue fever was developed during the largest dengue outbreak in Taiwan history in 2015. Currently, the NATs were performed by the TBSF to screen the presence of HBV, HCV and HIV using a mini-pool of eight samples from eight donors in Taiwan. Our results suggested that the use of a mini-pool (ten sera in a pool) seems acceptable in DENV RNA screening using real-time RT-PCR, since the results was similar to that of the screening using single serum of donors. The cost of the DENV RNA screening will be one-tenth using mini-pool strategy comparing to single serum screening.

## Supporting information

S1 Fig Annual dengue fever cases from 1987 to 2017 in Taiwan. Confirmed dengue cases in Taiwan since 1987 based on the data retrieved from the web-based notifiable diseases surveillance system maintained by the Taiwan CDC. Source of data: https://nidss.cdc.gov.tw/en/Default.aspx?op=4.
(TIF)

S2 Fig Distribution of dengue fever cases in 2015 in Taiwan. Confirmed dengue case numbers are shown in only the three southeastern cities that were the second-level administrative divisions with the highest levels of endemic dengue in Taiwan’s history. The gray line represents the Northern Tropic. This figure was generated using the map data described above and Quantum GIS v2.18.15 (QGIS Development Team, 2018. QGIS Geographic Information System. Open Source Geospatial Foundation. URL http://www.qgis.org/en/site/) using WGS84 (EPSG: 4326) as the default Coordinate Reference System (CRS) for datum transformations. Taiwan map data were retrieved from the Taiwan Geospatial One-Stop Portal developed by the Information Center of the Taiwan Ministry of The Interior and used under the Open Government Data License.
(TIF)

S1 Table Dengue seroprevalence in blood donors in different countries. (DOCX)

S1 File Supporting information-methods. (DOCX)
