# Supplementary figures and images for: Low frequency of asymptomatic dengue virus-infected donors in blood donor centers during the largest dengue outbreak in Taiwan
Source: PLoS One. 2018 Oct 8;13(10):e0205248. doi: 10.1371/journal.pone.0205248 (PMC6175512; doi:10.1371/journal.pone.0205248)

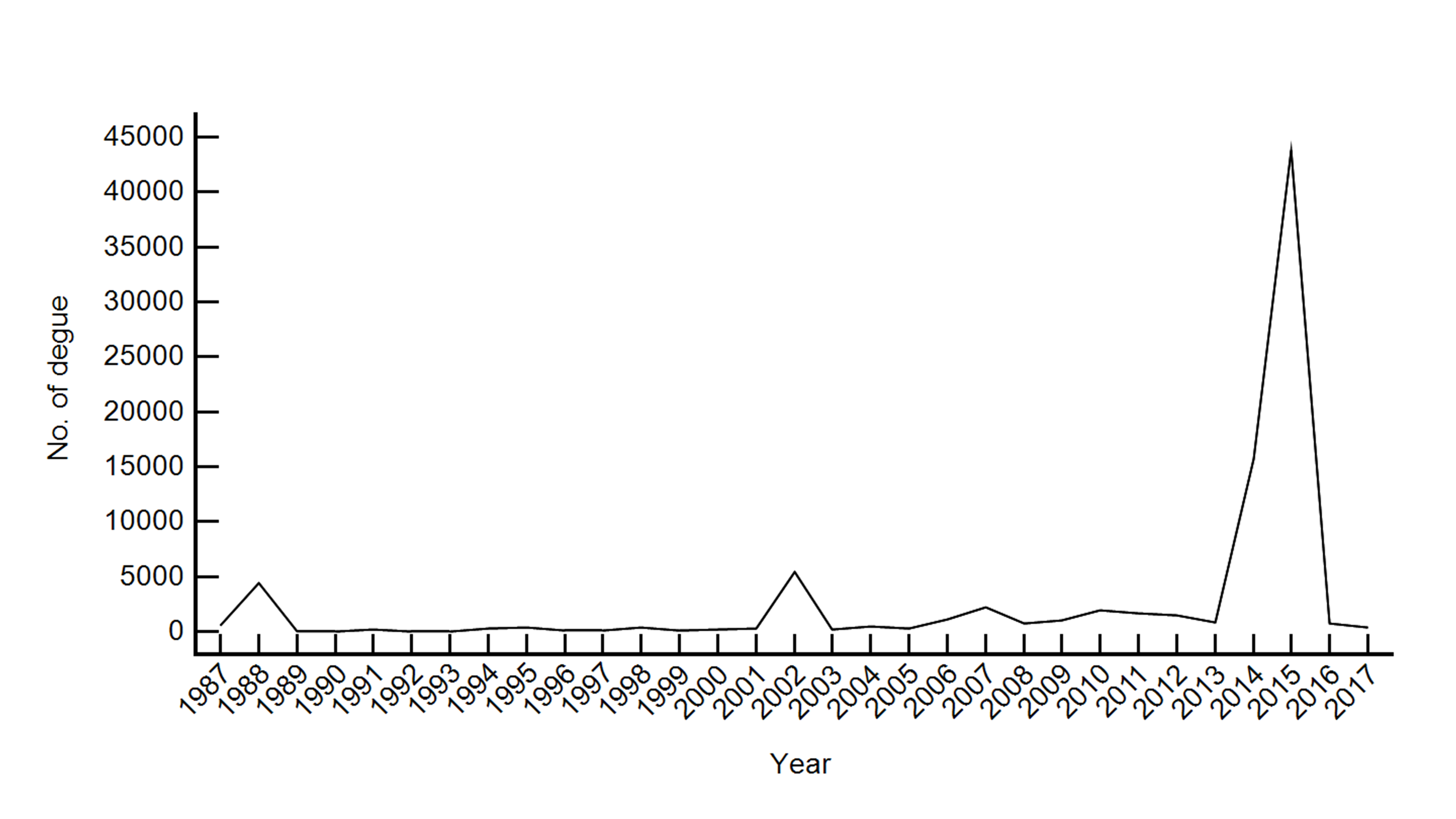

Supplement: S1 Fig — Confirmed dengue cases in Taiwan since 1987 based on the data retrieved from the web-based notifiable diseases surveillance system maintained by the Taiwan CDC. Source of data: https://nidss.cdc.gov.tw/en/Default.aspx?op=4. (TIF) [file pone.0205248.s001.TIF]

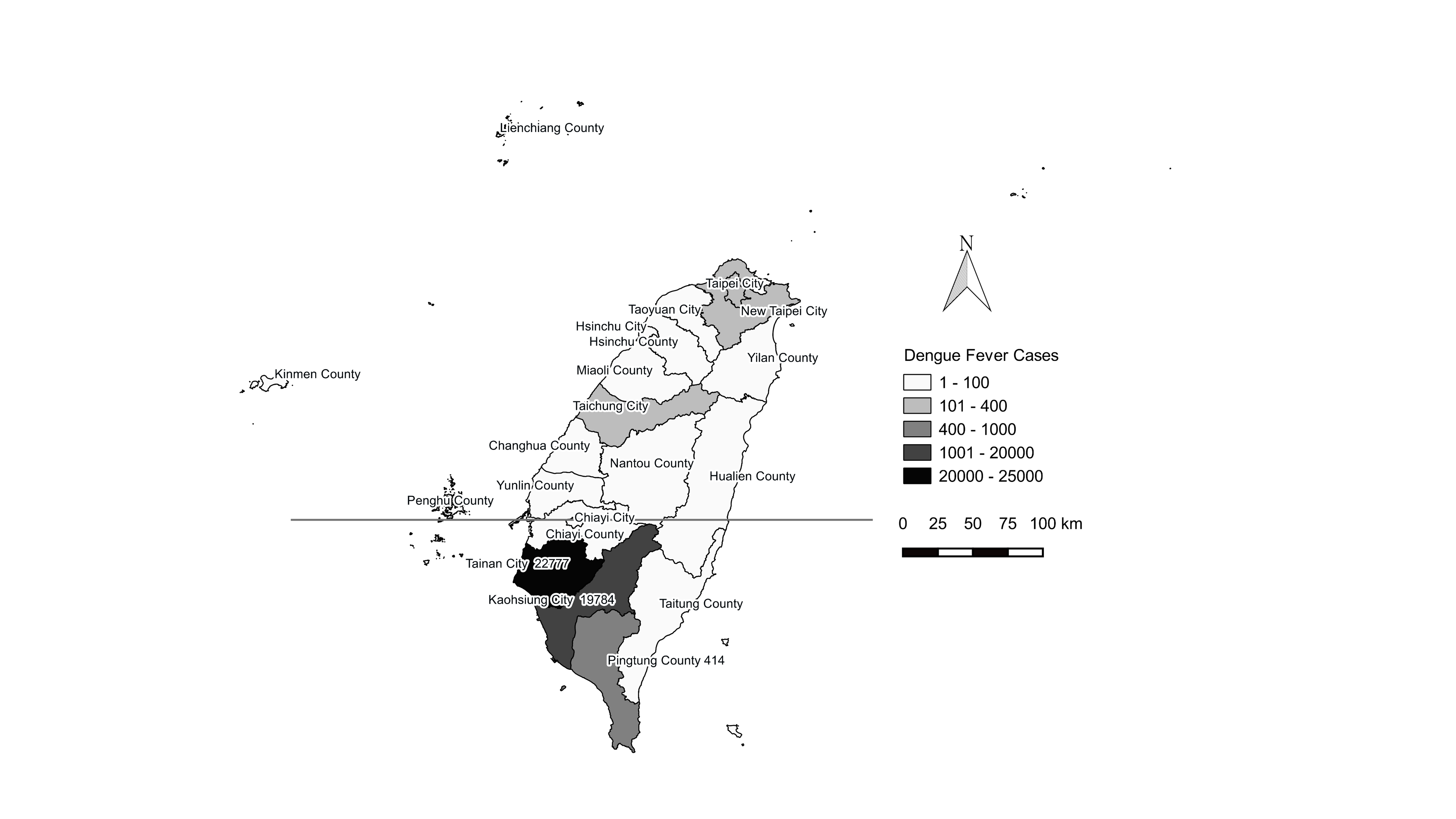

Supplement: S2 Fig — Confirmed dengue case numbers are shown in only the three southeastern cities that were the second-level administrative divisions with the highest levels of endemic dengue in Taiwan’s history. The gray line represents the Northern Tropic. This figure was generated using the map data described above and Quantum GIS v2.18.15 (QGIS Development Team, 2018. QGIS Geographic Information System. Open Source Geospatial Foundation. URL http://www.qgis.org/en/site/) using WGS84 (EPSG: 4326) as the default Coordinate Reference System (CRS) for datum transformations. Taiwan map data were retrieved from the Taiwan Geospatial One-Stop Portal developed by the Information Center of the Taiwan Ministry of The Interior and used under the Open Government Data License. (TIF) [file pone.0205248.s002.TIF]
