# Supplementary material for: Low frequency of asymptomatic dengue virus-infected donors in blood donor centers during the largest dengue outbreak in Taiwan
Source: PLoS One. 2018 Oct 8;13(10):e0205248. doi: 10.1371/journal.pone.0205248 (PMC6175512; doi:10.1371/journal.pone.0205248)
Supplement: S1 Table — (DOCX) [file pone.0205248.s003.docx]

**S1 Table. Dengue Seroprevalence in Blood Donors in Different Countries.**

| Country | Methodology | Sample Size | Target | Positive Case No. (%) | Serotype | Article Publication Year |
| --- | --- | --- | --- | --- | --- | --- |
| Honduras | TMA  ELISA | 2,994 | RNA  IgM, IgG | RNA: 9 (0.3%)  IgM: 2 (0.07%)  IgG: 4 (0.134%) | DENV-1/2/4 | [[14](#_ENREF_14)] Linnen et al. (2008) |
| Brazil | TMA | 4,858 | RNA | RNA: 3 (0.06) | DENV-1/3 |  |
| Australia | TMA | 5,879 | RNA | RNA: 0 (0%) | NA |  |
| Puerto Rico | TMA | 16,521 | RNA | RNA: 12 (0.07) | DENV-2/3 | [[15](#_ENREF_15)] Mohammed et al., 2008 |
|  | ELISA |  | IgM, IgG | IgM: 1 (0.0006%) |  |  |
|  |  |  |  | IgG: 9 (0.055%) |  |  |
| Brazil | RT-PCR | 500 | RNA | RNA: 2 (0.4%) | DENV-3 | [[39](#_ENREF_39)] Dias et al. (2012) |
| Australia  Queensland | ELISA | 5,453 | IgM, IgG | IgM: 12 (0.22%)  IgG: 9.43%* | NA | [[29](#_ENREF_29)] Faddy et al. (2013) |
| Carins |  | 2,416 |  | IgM: 8 (0.33%)  IgG: 7.18%* |  |  |
| Townsville |  | 3,037 |  | IgM: 4 (0.13%)  IgG: 11.48%* |  |  |
| Malaysia | ELISA | 360 | IgM, IgG | IgM: 15 (4.2%)  IgG: 141 (39.12%)  IgM/IgG: 10 (2.8%) | NA | [[30](#_ENREF_30)] Harif et al. (2014) |
| Singapore | ELISA | 3,995 | IgM, IgG | IgM: 2.83%  IgG: 52% | DENV-1/2/3/4 | [[31](#_ENREF_31)] Low et al. (2015) |
| India | ELISA | 1,709 | NS1 | NS1: 0 (0) | NA | [[40](#_ENREF_40)] Mangwana (2015) |
| Saudi Arabia | ELISA | 100 | NS1  IgM, IgG | NS1: 1 (1%)  IgM: 6 (6%)  IgG: 7 (7%) | NA | [[16](#_ENREF_16)] Ashshi (2015) |
| Brazil | TMA  ELISA | 16,241 | RNA  IgM, IgG | RNA: 87 (0.54%)  IgM: 2.8% to 8.8%†  IgG: 88.7%-90.9%† | DENV-4 | [[38](#_ENREF_38)] Busch et al. (2016) |
| India  Delhi | RT-PCR  ELISA | 200 | RNA  IgM, IgG | RNA: 0 (0%)  IgM: 27 (13.5%)  IgG: 116 (58%)  IgM/IgG: 25 (12.5%) | NA | [[32](#_ENREF_32)] Ranjan et al. (2016) |
| China  Guangxi | RT-PCR  ELISA | 1,685 | RNA  IgM, IgG | RNA: 0 (0%)  IgM: 6 (0.36%)  IgG: 7 (0.42%) | NA | [[34](#_ENREF_34)]Gao et al. (2017) |
| China Guangzhou | RT-PCR  ELISA | 3,000  1,500 | RNA  IgM, IgG | RNA: 2/3000  (0.007%) (IgM+/IgG-)  IgG: 51/1500 (3.4%) | NA | [[35](#_ENREF_35)] Liao et al. (2017) |
| Saudi Arabia | RT-PCR  ELISA | 910 | RNA  IgM, IgG | RNA: 50 (5.5%)  IgM: 50 (5.5%)  IgG: 335 (36.8%)  IgM/IgG: 38 (4.2%) | DENV-1/2/3/4 | [[33](#_ENREF_33)] Ashshi (2017) |
| Taiwan | RT-PCR  RDT & ELISA  RDT | 8,000 | RNA  IgM, IgG  NS1 | RNA: 1 (0.013%)  IgM: 17 (0.21%)  IgG: 13 (0.16%)  IgM/IgG: 13 (0.16%)  NS1: 0 (0%) | DENV-2 | This study |

*Only selected samples were analyzed for the presence of IgG.

†The results represent early and late epidemic samples.

NA: not available.

TMA: transcription-mediated amplification.
