# Supplementary material for: Low frequency of asymptomatic dengue virus-infected donors in blood donor centers during the largest dengue outbreak in Taiwan
Source: PLoS One. 2018 Oct 8;13(10):e0205248. doi: 10.1371/journal.pone.0205248 (PMC6175512; doi:10.1371/journal.pone.0205248)
Supplement: S1 File — (DOCX) [file pone.0205248.s004.docx]

**Methods**

**Sequencing**

The sequence of amplification products of the conventional serotype-specific RT-PCR was determined using the sequencing service provided by Mission Biotech company (Taipei City, Taiwan) using BigDye Terminator v3.1 Cycle Sequencing Kit in a 3730xl DNA Analyzer (Thermo Fisher Scientific Inc., Waltham, Massachusetts, USA).

**In vitro quantification of DENV RNA**

Quantification of DENV RNA in the culture supernatants were performed using Dengue Virus subtypes 1, 2, 3 and 4 genesig Standard Kit according to the manufacturer's instructions (Primerdesign Ltd, Camberley, UK).
